# 

**Published:** 1899-01

**Authors:** 


### Communicated.

CHICAGO, ILL., Dec. 28th, 1898.

The Chicaga Dental Society will, on Friday and Saturday, February 3rd and 4th, celebrate its thirty-fifth anniversary by holding a two day meeting for clinics, papers and discussions ending with a banquet Saturday night. A cordial invitation is extended to the profession to be present.

Printed programmes will be ready in January and may be had upon application.

JOSEPH W. WASSALL,
Sec'y., Com. of Arrangements.

### To Readers and Correspondents.

Communications intended for publication, and exchange journals and papers, should be addressed to the Editor of The American Journal of Dental Science, No. 845 N. Eutaw St. Baltimore, Md. All letters relating to the business of the Journal, or containing cash remittances, to be directed to Snowden & Cowman Mfg. Co., 9 W. Fayette Street, Baltimore, Md. Articles for publication should be received by the Editor at least two weeks previous to the first of the month on which the number in which it is designed for them to appear is issued.

☞ The American Journal of Dental Science will contain fifty pages of reading matter in each number, making a volume of about Six Hundred pages,

EXCLUSIVE OF ADVERTISEMENTS.

